# Determination of the protein quality of cooked Canadian pulses

**DOI:** 10.1002/fsn3.473

**Published:** 2017-06-20

**Authors:** Matthew G. Nosworthy, Jason Neufeld, Peter Frohlich, Gina Young, Linda Malcolmson, James D. House

**Affiliations:** ^1^ Department of Human Nutritional Sciences University of Manitoba Winnipeg MB Canada; ^2^ Canadian International Grains Institute Winnipeg MB Canada; ^3^ Richardson Centre for Functional Foods and Nutraceuticals University of Manitoba Winnipeg MB Canada; ^4^ Department of Food Science University of Manitoba Winnipeg MB Canada; ^5^ Canadian Centre for Agri‐Food Research in Health and Medicine and University of Manitoba Winnipeg MB Canada; ^6^ Department of Animal Science University of Manitoba Winnipeg MB Canada

**Keywords:** digestible indispensable amino acid score, protein digestibility‐corrected amino acid score, protein efficiency ratio, pulses

## Abstract

A study to determine the protein digestibility‐corrected amino acid score and protein efficiency ratio of nine different cooked Canadian pulse classes was conducted in support of the establishment of protein quality claims in Canada and the United States. Split green and yellow pea, whole green lentil, split red lentil, Kabuli chickpea, navy bean, pinto bean, light red kidney bean, and black bean were investigated. Protein digestibility‐corrected amino acid score (PDCAAS) and the protein efficiency ratio (PER) were determined using the appropriate rodent models. All pulses had high digestibility values, >70%, with PDCAAS values greater than 0.5, thereby qualifying as a quality protein in the United States, but only navy beans qualified as a good source of protein. All pulses except whole green lentils, split red lentils, and split green peas would qualify as sources of protein with protein ratings between 20 and 30.4 in Canada. These findings support the use of pulses as protein sources in the regulatory context of both the United States and Canada.

## 1 INTRODUCTION

Pulses constitute the dry, edible seeds from nonoilseed legumes, including beans, peas, chickpeas, and lentils. Pulses are generally characterized as rich sources of complex carbohydrates, vitamins, and minerals, including folate, potassium, and iron. Additionally, they contain significant amounts of protein (22%–24% by weight) and reflect important plant‐based sources of this macronutrient. Informing consumers that pulses constitute significant sources of dietary proteins can be accomplished through the use of protein content claims on food labels. However, evidence is needed to support any given claim made on a label. With respect to protein content claims, Canada, the United States, and almost all other nations have defined regulatory requirements. In Canada, the official method for determining the protein quality of a food is the Protein Rating system (Health Canada, 1981). This system relies on the use of the Protein Efficiency Ratio (PER), effectively a rat bioassay that measures weight gain per unit of protein consumed, using casein as a reference (Health Canada, 1981). The product of the PER and the amount of protein contained within a defined serving give rise to the Protein Rating. Foods with Protein Ratings between 20.0 and 39.9 qualify for a “Source of Protein” content claim, while those foods with a Protein Rating of 40.0 or greater qualify for an “Excellent Source of Protein” content claim.

Within the United States, protein quality is evaluated not by the PER, but by the 1991 WHO/FAO/UNU (FAO/WHO, 1991) established methodology entitled the Protein Digestibility‐Corrected Amino Acid Score (PDCAAS). For this methodology, the amino acid composition of a food protein is determined and the amino acid present in the lowest amount relative to a reference requirement pattern reflects the value of the amino acid score. Values less than 1.0 reflect the fact that at least one amino acid is limiting relative to the requirement pattern. If a food protein is completely digested and absorbed, then the amino acid score reflects the inherent limitations of a given protein for productive purposes. Most food proteins, particularly plant‐based food proteins, are not completely digestible. The PDCAAS reflects an attempt to measure the overall quality of a protein as the product of the digestibility of the protein and its amino acid score. Recently, a new method has been proposed for determining protein quality called the Digestible Indispensable Amino Acid Score (DIAAS) (FAO/WHO, 2013). This approach uses ileal digestibility rather than fecal, and considers the digestibility of individual amino acids instead of a single value; however, this method has yet to be officially adopted by any jurisdiction. Interestingly, the European Union does not use PER, PDCAAS, or DIAAS for its regulation of protein content claims, rather if a food product contributes 12% of its total energy via protein it is considered a ‘source of protein’; if the contribution of protein to total energy is 20% that product has ‘high protein’ (European Commission, 2017).

Pulse crops contain antinutritive factors that influence protein digestibility and nutrient availability which, in turn, alters protein quality (Bhatty, Christison, & Centre, 1984; Candela, Astiasaran, & Belli, 1997; Gupta, 1987; Hahn, Rooney, & Earp, 1984; Oomah, Caspar, Malcolmson, & Bellido, 2011). When cooked, however, beans, chickpeas, peas, and lentils demonstrate reduced trypsin inhibitor activity and lower tannin concentration (Wang, Hatcher, & Gawalko, 2008; Wang, Hatcher, Toews, & Gawalko, 2009; Wang, Hatcher, Tyler, Toews, & Gawalko, 2010). Cooking has also been shown to increase the protein content of kidney beans, faba beans, and chickpeas (Candela et al., 1997; Fernandez, Lopez‐Jurado, Aranda, & Urbano, 1996; Wang et al., 2010), possibly through the removal of carbohydrates (Savage & Thompson, 1993; Vidal‐Valverde, Frias, & Valverde, 1992). Interestingly, cooking has also been shown to increase the concentration of essential amino acids in kidney beans, faba beans, and chickpeas compared to uncooked pulses (Alajaji & El‐Adawy, 2006; Bhatty et al., 1984; Khattab, Arntfield, & Nyachoti, 2009). Reduction in antinutritive factors and alteration of the amino acid profile of a protein source through cooking would alter both PER and PDCAAS values, thereby impacting protein content claims in North America. Additionally, cooking may influence the serving size via changes in the final moisture content, and thus density, of the cooked pulses. This can also impact the potential to reach specific protein content claims.

This study was conducted to determine the Protein Rating and the PDCAAS of cooked Canadian pulses in support of the establishment of protein content claims in both Canada and the United States Although not currently utilized for regulation of protein content claims, DIAAS was also calculated for comparison with PDCAAS values using true protein digestibility as an interim measure proposed by the FAO (FAO/WHO, 1991).

## 2 MATERIALS AND METHODS

All procedures were approved by the Institutional Animal Care Committee in accordance with the guidelines of the Canadian Council on Animal Care (Protocol Number F2012‐035).

### 2.1 Sourcing of pulse samples

For the current study, composite pulse samples were prepared from individual lots collected from several processors across Canada from the 2010 cropping year. Composite samples were prepared by the Canadian International Grains Institute by blending an equal amount of each processors sample for 5 min using a Hobart mixer (Model HL 200). A description of the pulse samples is presented in Table 1.

**Table 1:** Description of the pulse market classes selected for protein quality evaluation

Pulse type	Market class	Scientific name	Level of processing	Crop year
Pea	Green	*Pisum sativum*	Dehulled, split	2010
Pea	Yellow	*Lathyrus aphaca*	Dehulled, split	2010
Lentil	Green	*Lens culinaris*	n/a	2010
Lentil	Red	*Lens culinaris*	Dehulled, split	2010
Chickpea	Kabuli	*Cicer arietinum*	n/a	2010
Bean	Navy	*Phaseolus vulgaris*	n/a	2010
Bean	Pinto	*Phaseolus vulgaris*	n/a	2010
Bean	Kidney	*Phaseolus vulgaris*	n/a	2010
Bean	Black	*Phaseolus vulgaris*	n/a	2010

### 2.2 Cooking of pulse samples

All beans and chickpeas were soaked for 16 hr prior to cooking. The lentils and split peas were cooked without soaking. Cooking times for the pulses are listed in Table 2. For chickpea, navy bean, black bean, kidney bean, and pinto bean cooking times were determined via an automated Mattson cooker (Wang & Daun, 2005). In brief, plunger weight was adjusted according to market class and each tip placed onto one seed of the appropriate market class. The loaded apparatus was then incubated in boiling water until all plungers had dropped. Cooking time was defined as the time required for 80% of the plungers to penetrate the seeds. For green lentil, split red lentil, split green, and split yellow pea samples cooking times were determined using a modified tactile method (Reyes‐Moreno, Paredes‐López, & Gonzalez, 1993). Briefly, 100 g of sample was added to 500 ml of gently boiling deionized water. After a predetermined time, 8 min for split pea, 10 min for whole lentil, and 6 min for split lentil samples were removed from the water and squeezed between thumb and forefinger. The point at which 4 out of 5 seeds had little to no resistance was considered as the cooking time. Following cooking, samples were rinsed 2× with 3L of cold tap water and freeze‐dried prior to biological and chemical analyses.

**Table 2:** Cooking times of nine samples of Canadian Pulses as determined by a modified tactile method^a^ or the Automated Mattson Cooker^b^

Pulse type	CT	CT	AVG
Split green pea^a^	34	34	34
Split yellow pea^a^	37	37	37
Green lentil^a^	26	26	26
Split red lentil^a^	12	12	12
Kabuli chickpea^b^	23.2	22.2	22.7
Navy bean^b^	19.4	17.7	18.6
Pinto bean^b^	18.1	20.2	19.2
Light red kidney bean^b^	25.3	23.7	24.5
Black bean^b^	18.2	18.7	18.5

CT, cooking time (min); AVG, average cooking time (min).

### 2.3 Analytical procedures

Prior to analysis, all samples were ground in a hand‐held electric mill. For all samples, percent crude protein (CP; N × 6.25) was determined through the use of a LECO CNS‐2000 Nitrogen Analyzer (LECO Corporation, St Joseph MI., U.S.A., Model No. 602‐00‐500), percent dry matter (DM), and ash were determined according to standard procedures (AOAC International, 1995). The percent crude fat was determined by extracting crude fat into hexane and by gravimetrics (AOAC International, 1995). The amino acid contents of the samples were determined by acid hydrolysis using the AOAC Official Method 982.30 (AOAC International, 1995). Methionine and cysteine were determined by the performic acid oxidized hydrolysis procedure, and tryptophan was determined using alkaline hydrolysis (AOAC International, 1995).

### 2.4 Protein digestibility‐corrected amino acid score determination

A rat bioassay, as described previously, was used to determine the PDCAAS of the pulse samples (AOAC International, 1995). Amino acid ratios for the samples and the reference protein casein were derived by dividing each essential amino acids' relative abundance in a pulse or casein test protein, expressed in milligrams of amino acid per gram of test protein, by the relative abundance of the same amino acid in the protein reference pattern adopted by the Food and Agriculture Organization of the United Nations (FAO) and the World Health Organization. The reference pattern used was those outlined in the 1991 FAO/WHO/UNU report (FAO/WHO, 1991). Amino acid scores were determined by selecting the value of the amino acid with the lowest ratio. True protein digestibility was determined using the AOAC Official Method 991.29 rat bioassay, using casein as a reference standard, and correcting for endogenous protein losses using a protein‐free diet (AOAC International, 1995). All test articles were ground to pass through a 2 mm screen prior to preparation of the test diets. Diets were formulated to contain 10% protein, supplied by the test pulse sample, 10% total fat (total of residual pulse fat and supplemental corn oil), and 5% cellulose with the remaining energy derived from corn starch. Vitamins and minerals (AIN‐93 formulations; Harlan Teklad, Madison, WI) were added to diets to meet the micronutrient requirements of laboratory rats. Male weanling laboratory rats (*n *=* *8–10 per treatment; initial weight 70 g) were individually housed in suspended wire‐bottomed cages, with absorbent paper placed underneath. Water was available for ad libitum consumption. Feed was restricted to a maximum of 15 g/day over a 4‐day acclimation period followed by a 5‐day balance period, during which daily feed intake was calculated. Total fecal output was collected during the balance period, air‐dried, and analyzed for its dry matter and nitrogen content. True protein digestibility (TPD%) was calculated as follows where nitrogen intake and fecal nitrogen loss represent the product of food intake or fecal weights and their respective nitrogen values:

$$\begin{array}{cc} \mathrm{TPD}\% & \;=\;((\mathrm{Nitrogen}\;\mathrm{Intake}\;-\;(\mathrm{Fecal}\;\mathrm{Nitrogen}\;\mathrm{Loss}\;-\\ & \mathrm{Metabolic}\;\mathrm{Nitrogen}\;\mathrm{Loss}))/\mathrm{Nitrogen}\;\mathrm{Intake})\;\times \;100 \end{array}$$

The value for metabolic nitrogen loss was determined as the amount of fecal nitrogen produced by rats consuming a protein‐free diet. The PDCAAS was calculated as the product of the amino acid score and TPD%. For the purposes of establishing a protein content claim in the United States, the products of the protein content of a 90 g representative serving size and the PDCAAS of each pulse were compared to the daily reference value (DRV) of 50 g of protein. Values between 10 and 19.9% of the DRV constitute “Good Sources” of protein.

### 2.5 Protein efficiency ratio

Protein Efficiency Ratio (PER) values were determined over a 28‐day growth period for rats consuming feed ad libitum (Health Canada, 1981). For the current study, the 28‐day growth period included the 9‐day protein digestibility study period. Rat weights were recorded throughout the acclimation and balance periods, and feed intake was recorded throughout the study. The Protein Efficiency Ratio was calculated as the amount of weight gain (g) divided by the amount of protein (g) consumed over 28 days. Values were adjusted to a standardized 2.5 PER value for the reference casein. For the purposes of establishing a protein content claim in Canada, the protein ratings for standard reference serving sizes for cooked pulses (250 ml) were calculated as the product of the Adjusted PER and the amount of protein contained in the reference serving (g). Protein ratings between 20 and 39.9 qualify for a “Source of Protein” claim, while values of 40 or greater qualify for an “Excellent Source of Protein Claim” (Health Canada, 1981).

### 2.6 Digestible indispensable amino acid score (DIAAS)

Digestible indispensable amino acid score (DIAAS) was calculated using the amino acid reference pattern for children aged 6 months to 3 years which was used in conjunction with the following equation (FAO/WHO, 2013):

$$\begin{array}{cc} \mathrm{DIAAS}\% & \;=\;100\;\times \;[(\mathrm{mg}\;\mathrm{of}\;\mathrm{digestible}\;\mathrm{dietary}\;\mathrm{indispensable}\\ & \mathrm{amino}\;\mathrm{acid}\;\mathrm{in}\;1g\;\mathrm{of}\;\mathrm{the}\;\mathrm{dietary}\;\mathrm{protein})/(\mathrm{mg}\;\mathrm{of}\;\mathrm{the}\;\mathrm{same}\\ & \mathrm{dietary}\;\;\mathrm{indispensable}\;\mathrm{amino}\;\mathrm{acid}\;\mathrm{in}\;1g\;\mathrm{of}\;\mathrm{the}\;\mathrm{reference}\;\mathrm{protein})] \end{array}$$

In this study fecal nitrogen digestibility calculated for PDCAAS was used to determine DIAAS rather than ileal digestibility. Although the FAO/WHO recommends that digestibility should be based on the true ileal digestibility of the individual amino acids, there is acknowledgment that until a dataset of true ileal digestibility is developed fecal crude digestibility should be used for the determination of DIAAS values (FAO/WHO, 2013).

## 3 RESULTS AND DISCUSSION

### 3.1 Proximate analysis and amino acid composition of cooked Canadian pulses

The data for percentage crude protein (%CP) and crude fat (%Fat) content of the various pulse classes are provided in Table 3 (dry matter basis), along with the amino acid composition of each pulse class being investigated. The protein content of the cooked pulses ranged from 21.92% for chickpea to 29.51% for split red lentil, which is similar to previously reported values of raw pulse protein content with chickpeas averaging 23%, peas 22.3%, beans 23%, and lentils 28.3% (Akibode & Maredia, 2012; El‐Adawy, 2000; Grusak, 2009n.d.; Tzitzikas, Vincken, de Groot, Gruppen, & Visser, 2006). When comparing the determined protein contents to the respective energy values for each pulse studied (derived from the USDA Nutrient Database, 2016), all of the pulses provided greater than 20% of the energy as protein (range 22% to 31% of total calories). As such, all of the studied pulses would qualify as “excellent sources of protein” under the system employed in the European Union. The fat content of the cooked pulses ranged from 1.19%, for split red lentil, to 6.89%, chickpea. Considering the fat content of raw pulses, lentil 1.06%, beans 1.3%, peas 0.4%, chickpea 6.4% (USDA, 2016), on average, all cooked pulses had a higher fat content than raw flours with peas having the largest increase, from 0.4% in uncooked pulses to 1.32% after cooking. The moisture contents (100 ‐ %DM) of the cooked pulse products ranged from a low of 64.28% for navy beans to a high of 75.25% for split red lentils. In general, the lentils and peas had higher moisture content (approx. 72%–75%) as compared to the beans and chickpeas (approx. 65%). Given the fact that serving sizes are expressed on a wet weight basis, the moisture content of the pulses can influence the grams of protein present in a given volume of a serving. Due to displacement, the moisture content will have a significant impact on the protein rating of a cooked pulse. By standardizing the cooking times through the use of automated Mattson cooker procedure, this study sought to achieve a level of “doneness” that reflected those typically encountered in consumer settings. As such, the density of the pulses, and thus the serving sizes, likely reflects those relevant for protein content claims at the consumer level. However, further opportunity exists to examine the impact of cooking methods on moisture content and, ultimately, the density calculations relative to standard serving sizes.

**Table 3:** Proximate composition and amino acid composition of cooked Canadian pulses (g/100 g on a Dry matter basis)

	Nutrient composition (g/100 g Dry matter basis)																			
Sample ID	%Fat	%CP	ASP	THR	SER	GLU	PRO	GLY	ALA	CYS	VAL	MET	ILE	LEU	TYR	PHE	HIS	LYS	ARG	TRP
Red kidney beans	1.64	23.94	2.85	1.05	1.56	3.62	1.00	0.98	1.06	0.18	0.96	0.24	0.79	1.80	0.65	1.24	0.66	1.61	1.15	0.22
Navy beans	1.87	24.52	2.89	1.10	1.56	3.43	1.02	1.01	1.08	0.24	1.14	0.30	0.94	1.94	0.70	1.40	0.67	1.70	1.28	0.23
Whole green lentils	1.39	26.27	3.37	1.11	1.50	4.84	1.25	1.24	1.27	0.26	1.15	0.21	1.01	2.12	0.85	1.38	0.70	2.13	2.25	0.21
Split red lentils	1.19	29.51	3.71	1.23	1.80	5.18	1.35	1.27	1.37	0.22	1.36	0.22	1.18	2.48	0.92	1.63	0.80	2.21	2.40	0.26
Split yellow peas	1.27	25.26	2.86	0.96	1.25	4.08	1.04	1.08	1.09	0.31	1.10	0.26	0.98	1.84	0.73	1.19	0.61	1.82	1.93	0.20
Split green peas	1.36	26.24	3.13	1.01	1.53	4.46	1.17	1.12	1.18	0.20	1.04	0.19	0.87	1.96	0.69	1.31	0.65	1.85	1.89	0.26
Black beans	2.01	23.95	3.23	1.26	1.78	3.90	1.11	1.12	1.21	0.21	1.17	0.25	1.00	2.12	0.78	1.43	0.73	1.81	1.41	0.25
Chickpeas	6.89	21.91	2.89	0.89	1.29	4.01	1.03	0.98	1.05	0.29	1.06	0.30	1.00	1.85	0.62	1.44	0.64	1.62	2.09	0.15
Pinto beans	1.84	22.68	2.84	1.06	1.54	3.51	0.98	1.00	1.07	0.21	1.04	0.27	0.90	1.90	0.70	1.27	0.67	1.66	1.22	0.19
Casein	0.00	86.78	6.90	4.47	5.61	21.83	11.60	1.78	2.93	0.68	5.64	2.65	4.50	9.23	5.49	4.99	2.55	7.51	2.79	1.00

% Fat, % crude fat, determined by solvent extraction; %CP, % crude protein determined by LECO. ASP, aspartate; THR, threonine; SER, serine; GLU, Glutamate; PRO, proline; GLY, glycine; ALA, alanine; CYS, cysteine (determined by oxidation method); VAL, valine; MET, methionine (determined by oxidation method); ILE, isoleucine; LEU, leucine; TYR, tyrosine; PHE, phenylalanine; HIS, histidine; LYS, lysine; ARG, arginine; TRP, tryptophan (determined via alkaline hydrolysis).

### 3.2 Amino acid scores of cooked Canadian pulses

The amino acid scores were calculated according to the 1991 FAO reference pattern (Table 4). Using the 1991 Reference Pattern, on the basis of the lowest ratios observed, the sulfur amino acids, methionine and cysteine, were limiting for red kidney beans, whole green lentils, split red lentils, split green peas, and black beans. Alternatively, the amino acid tryptophan was limiting for navy beans, split yellow peas, chickpeas, and pinto beans. Other groups have found similar results when determining limiting amino acids in pulses (Jackson, 2009; Sarwar & Peace, 1986; Wu et al., 1996). The amino acid scores of the cooked pulses ranged from 0.59 (split red lentils and split green peas) to a high of 0.83 for navy beans.

**Table 4:** Protein digestibility corrected amino acid scores of cooked Canadian pulses (1991 reference pattern)

	Amino Acid Score											
	HIS	ILE	LEU	LYS	M+C^a^	P+T^b^	THR	TRP	VAL	AA Score	TPD (%)	PDCAAS
Red kidney beans	1.46	1.17	1.14	1.16	**0.70**	1.25	1.29	0.83	1.15	0.698	78.60	0.549
Navy beans	1.44	1.37	1.20	1.20	0.88	1.36	1.32	**0.83**	1.33	0.834	79.96	0.667
Whole green lentils	1.41	1.38	1.23	1.40	**0.71**	1.35	1.24	0.72	1.25	0.714	87.89	0.628
Split red lentils	1.42	1.42	1.27	1.29	**0.59**	1.37	1.23	0.80	1.32	0.594	90.60	0.538
Split yellow peas	1.26	1.38	1.10	1.25	0.90	1.21	1.11	**0.73**	1.25	0.731	87.94	0.643
Split green peas	1.31	1.19	1.13	1.22	**0.59**	1.21	1.13	0.89	1.13	0.587	85.15	0.500
Black beans	1.61	1.49	1.34	1.30	**0.76**	1.46	1.54	0.95	1.40	0.763	69.99	0.534
Chickpeas	1.54	1.63	1.28	1.27	1.08	1.50	1.19	**0.61**	1.39	0.610	85.02	0.519
Pinto beans	1.55	1.42	1.27	1.26	0.85	1.38	1.38	**0.77**	1.31	0.774	76.23	0.590
Casein	1.55	1.85	1.61	1.49	1.53	1.92	1.52	**1.05**	1.86	1.049	96.59	1.000

Bolded values reflect first limiting amino acid. TPD, True Protein Digestibility; PDCAAS, Protein Digestibility‐Corrected Amino Acid Score. PDCAAS is calculated as the product of AAS and %TPD.

a M+C  =  Methionine + Cysteine.

b P+T = Phenylalanine + Tyrosine.

### 3.3 True protein digestibility & PDCAAS

True protein digestibility values are presented in Table 4 and reflect the amount of nitrogen digested by rats during a bioassay, with corrections for endogenous nitrogen losses. In general, the protein in cooked pulses is highly digestible, with values being 70% or greater. True protein digestibility (TPD %) was lowest for black beans (70%), and highest for the split red lentils (90.6%). Previous investigation into the digestibility of autoclaved pulse flour found similar digestibilities of peas 88%, common beans 79% (average), lentils 85%, and black beans 72% (FAO/WHO, 1991)_._ The lower value observed in black beans may be related to the phenolic fractions present in these beans, or other factors not yet recognized. With respect to chickpea, raw seed has been found to have a digestibility of 72% which increases to 84% after cooking (Clemente, Shnchez‐vioque, Vioque, Bautistab, & Millin, 1998), similar to the results of this study. Pulses contain a wide variety of antinutritional factors that reduce digestibility and nutrient availability including proteolytic inhibitors (Gupta, 1987; Oomah et al., 2011) and tannins (Hahn et al., 1984). Cooking has been shown to reduce trypsin inhibitor activity and tannin concentrations (Wang et al., 2008, 2009, 2010), which contribute to increased protein digestibility.

The product of the true protein digestibility and the amino acid score provides the PDCAAS values, and these are presented in Table 4. In general, the PDCAAS values were 0.5 or greater, with the highest value observed for navy beans, 0.67. These data are similar to the PDCAAS values of autoclaved pinto beans 0.62 and black beans 0.53. Conversely, green lentils had a higher PDCAAS value than the average lentil 0.63 versus 0.51 (FAO/WHO, 1991), split green peas were lower 0.50 versus 0.65 (FAO/WHO, 1991), and chickpea flour was higher 0.52 versus 0.44 (Tavano, da Silva, Demonte, & Neves, 2008). These differences are potentially due to differences in preparatory method, cooking compared to autoclaving, however, varietal differences may also have had an effect.

**Table 5:** Digestible indispensable amino acid values of cooked Canadian pulses

	HIS	ILE	LEU	LYS	M+C^a^	P+T^b^	THR	TRP	VAL	DIAAS
Red kidney beans	1.09	0.81	0.9	0.93	**0.51**	1.19	1.12	0.85	0.74	0.51
Navy beans	1.1	0.96	0.96	0.98	**0.65**	1.32	1.15	0.86	0.87	0.65
Whole green lentils	1.18	1.06	1.08	1.25	**0.58**	1.43	1.2	0.82	0.9	0.58
Split red lentils	1.23	1.13	1.16	1.2	**0.5**	1.51	1.22	0.94	0.98	0.50
Split yellow peas	1.06	1.06	0.97	1.11	**0.73**	1.29	1.07	0.83	0.89	0.73
Split green peas	1.05	0.88	0.96	1.05	**0.46**	1.24	1.05	0.98	0.78	0.46
Black beans	1.07	0.91	0.94	0.93	**0.49**	1.24	1.18	0.86	0.8	0.49
Chickpeas	1.24	1.21	1.09	1.1	0.85	1.54	1.11	**0.67**	0.96	0.67
Pinto beans	1.12	0.95	0.96	0.98	**0.6**	1.27	1.15	0.76	0.81	0.60
Casein	1.42	1.56	1.56	1.47	1.37	2.24	1.61	**1.31**	1.46	1.31

DIAAS was calculated using true protein digestibility. Bolded values reflect first limiting amino acid. DIAAS, Digestible Indispensable Amino Acid Score. DIAAS values are determined by the value of the first limiting amino acid.

a M+C = Methionine + Cysteine.

b P+T = Phenylalanine + Tyrosine.

Under U.S. labeling regulations, a protein must have a PDCAAS value greater than 0.2 (20%) in order to qualify as a quality protein for noninfant foods, and greater than 0.4 (40%) to qualify as a quality protein for foods intended for infants (FAO/WHO, 2013). In order to make a protein content claim in the United States, foods must first meet the definition of a quality protein and all of the pulses tested met these criteria (Table 6). Additionally, the protein provided in a standard 90 g serving of pulses, corrected for the PDCAAS (i.e., protein in grams × PDCAAS), must be between 10.0 and 19.9% of the daily reference value of 50 g of protein in order to qualify for a “Good Source of Protein” claim. Foods with greater than 20% of the daily reference qualify for an “Excellent Source of Protein” content claim. For the pulses tested only navy beans would qualify as a “Good Source of Protein” in the United States. A limitation in the U.S. labeling system primarily relates to the defined serving size of pulses at 90 g. Using density values from the Canadian Nutrient File (Health Canada, 2016), the weights of a 175 ml serving size of the pulses studied were 121 g or greater. Clearly, the larger the serving size, the more protein present in the serving which then influences the extent of the protein content claim that can be made.

**Table 6:** Percentage of the daily reference values (50 g) provided by a 90 g serving of cooked Canadian pulses and Adjusted Protein Efficiency Ratios and Protein Ratings (FDA and Health Canada regulatory requirements)

		% Daily reference values – U.S. regulatory system			Protein efficiency ratios & protein ratings – Canadian regulatory system			
	Protein (CP) (g/100 g)	PDCAAS	Corrected CP per serving	%Daily reference value	Adj. PER	g/250 ml	CP (g/250 ml serving)	Protein rating (250 ml serving)
Red kidney beans	8.27	0.549	4.09	8.17	1.55	187.00	15.47	**23.98**
Navy beans	8.76	0.667	5.26	**10.52**	1.51	192.30	16.84	**25.43**
Whole green lentils	6.72	0.628	3.80	7.60	1.30	209.20	14.07	18.29
Split red lentils	7.30	0.538	3.54	7.07	0.98	255.80	18.68	18.31
Split yellow peas	6.81	0.643	3.94	7.87	1.42	207.10	14.09	**20.01**
Split Green Peas	7.39	0.500	3.33	6.65	0.86	207.10	15.31	13.17
Black beans	8.39	0.534	4.03	8.07	1.61	181.70	15.25	**24.55**
Chickpeas	7.57	0.519	3.53	7.07	2.32	173.30	13.12	**30.44**
Pinto beans	7.85	0.590	4.17	8.33	1.64	180.70	14.19	**23.27**

Bolded values qualify for “Good Source of Protein” claim in United States and Canada, respectively. The reference serving size used for pulses in the United States was 90 g and protein content is provided on an as is basis.

The digestible indispensable amino acid score (DIAAS) was initially recommended by the FAO/WHO for the determination of protein quality in a report released in 2013 (FAO/WHO, 2013). Although this method suggests the use of ileal amino acid digestibility rather than fecal digestibility, the FAO/WHO working group acknowledged that using fecal digestibility in conjunction with the updated amino acid pattern would be acceptable (FAO/WHO, 2013). In these samples, the DIAAS values range from 0.46 (split green peas) to 0.73 (split yellow peas) (Table 5). Chickpea, however, has a higher DIAAS value than the PDCAAS (0.67 vs. 0.52) due to its high methionine content and the lowering of the tryptophan requirement from 11 mg/g protein for PDCAAS in 1991 to 8.5 mg/g protein for DIAAS. In the 2013 FAO/WHO report, it was recommended that no nutrition claim be allowed for protein sources with a DIAAS less than 0.75. This fact is at odds with current dietary guidelines as cooked pulses are positioned as protein sources in both Canada's Food Guide and the MyPlate system while this study demonstrates that no cooked pulses have a DIAAS value passing that threshold. For this reason the use of a cut‐off of 0.75 for DIAAS for the establishment of protein content claims should continue to be evaluated.

### 3.4 Protein efficiency ratios and protein ratings

Within Canada, the official method for determining the protein quality of foods is the Protein Efficiency Ratio (PER) (Health Canada, 1981). The PER is a rat bioassay method in which rats consume a semipurified diet where the protein of interest represents the sole source of protein (present at 10% of the diet). In order to calculate PER both protein consumption and weight gain are monitored over a 28‐day period with the PER being calculated as the total weight gain divided by the total protein intake. In order to provide consistency between studies, casein is used as a reference protein, and all PER values are adjusted to a common PER for casein of 2.5. The data for the adjusted PER values for all cooked pulses are presented in Table 6. The adjusted PER values for the pulses ranged from a low of 0.86 for Split Green Peas to a high of 2.32 for Chickpeas. In order to successfully obtain a Canadian protein content claim, a serving of a given food must have a Protein Rating of 20.0–39.9 to qualify as a “Source of Protein”. Protein Ratings above 40.0 qualify for an “Excellent Source of Protein” claim. The Protein Ratings are calculated as the product of the Adjusted PER and the amount of crude protein (grams of N × 6.25) in a representative serving of the food. The data given in Table 6 have been derived using a serving size of 250 ml. Using this serving size, all pulses, with the exception of Whole Green Lentils, Split Red Lentils, and Split Green Peas, would qualify as “Sources of Protein”, with Protein Ratings between 20 and 30.4.

## 4 CONCLUSIONS

In summary, data are presented in support of the establishment of protein content claims in Canada and the U.S. Cooked Canadian Pulses contain protein that is highly digestible (typically greater than 80%). Limiting amino acids within the proteins will depend on the choice of reference amino acid pattern, but typically the sulfur amino acids or tryptophan is the first limiting amino acid. Protein Rating values presented support the use of “Source of Protein” content claims for most pulses. The use of the PDCAAS will support the establishment of “Good Source of Protein” content claims in the United States for Navy Beans, Split Yellow Peas, Chickpeas, and Pinto Beans.

## CONFLICTS OF INTEREST

The authors declare that they have no conflicts of interest.

## References

[fsn3473-bib-0001] Akibode, S. , & Maredia, M . (2012). Global and regional trends in production, trade and consumption of food legume crops. Retrieved from http://impact.cgiar.org/sites/default/files/images/Legumetrendsv2.pdf

[fsn3473-bib-0002] Alajaji, S. A. , & El‐Adawy, T. A. (2006). Nutritional composition of chickpea (Cicer arietinum L.) as affected by microwave cooking and other traditional cooking methods. Journal of Food Composition and Analysis, 19, 806–812.

[fsn3473-bib-0003] AOAC International . (1995). Official methods of Analysis of AOAC International. 16^th^ Edition. Arlington VA: AOAC International.

[fsn3473-bib-0004] Bhatty, R. S. , Christison, G. I. , & Centre, C. D. (1984). Laboratory analysis Proximate composition of the three meals, protein concentrates and isolates. Plant Foods for Human Nutrition, 34, 41–51.

[fsn3473-bib-0005] Candela, M. , Astiasaran, I. , & Belli, J. (1997). Cooking and warm‐holding: Effect on general composition and amino acids of kidney beans (Phaseolus vulgaris), chickpeas (Cicer arietinum), and lentils (Lens culinaris). Journal of Agricultural and Food Chemistry, 45, 4763–4767.

[fsn3473-bib-0006] Clemente, A. , Shnchez‐vioque, R. , Vioque, J. , Bautistab, J. , & Millin, F. (1998). Effect of cooking on protein quality of chickpea (Cicer arietinrcm) seeds. Food Chemistry, 62(1), 1–6.

[fsn3473-bib-0007] El‐Adawy, T. (2000). Functional properties and nutritional quality of acetylated and succinylated mung bean protein isolate. Food Chemistry., 70, 83–91.

[fsn3473-bib-0008] European Commission . (2017) Nutrition Claims. Retrieved from http://ec.europa.eu/food/safety/labelling_nutrition/claims/nutrition_claims_en.

[fsn3473-bib-0009] FAO/WHO . (1991). Protein quality evaluation. Report of the Joint FAO/WHO Expert Consultation. Food and Nutrition Paper No. 51.

[fsn3473-bib-0010] FAO/WHO . (2013). Dietary protein quality evaluation in human nutrition. Report of an FAO Expert Consultation. Food and Nutrition Paper No.92 26369006

[fsn3473-bib-0011] Fernandez, M. , Lopez‐Jurado, M. , Aranda, P. , & Urbano, G. (1996). Nutritional assessment of raw and processed faba bean (Vicia faba L.) cultivar major in growing rats. Journal of Agricultural and Food Chemistry, 44, 2766–2772.

[fsn3473-bib-0012] Grusak, M. A . (2009). Nutritional and health‐beneficial quality In Erskine W., Muehlbauer F. J., Sarker A., & Sharma B. (eds.), The lentil: Botany, production and uses (pp. 368–390). Wallingford: CABI.

[fsn3473-bib-0013] Gupta, Y. P. (1987). Anti‐nutritional and toxic factors in food legumes: A review. Plant Foods for Human Nutrition, 37, 201–228. 2853348 10.1007/BF01091786

[fsn3473-bib-0014] Hahn, D. , Rooney, L. , & Earp, C . (1984). Tannins and phenols of sorghum. Cereal Foods World (USA). Retrieved from http://agris.fao.org/agris-search/search.do?recordID=US8602509

[fsn3473-bib-0015] Health Canada . (1981). Determination of Protein Rating FO‐1. Retreived from http://www.hc-sc.gc.ca/fn-an/alt_formats/hpfb-dgpsa/pdf/res-rech/fo-1-eng.pdf

[fsn3473-bib-0016] Health Canada . (2016). Canadian Nutrient File Search Engine Online. Retreived from https://food-nutrition.canada.ca/cnf-fce/index-eng.jsp

[fsn3473-bib-0017] Jackson, J. (2009). Protein nutritional quality of cowpea and navy bean residue fractions. African Journal of Food, Agriculture, Nutrition and Development, 9, 764–778.

[fsn3473-bib-0018] Khattab, R. Y. , Arntfield, S. D. , & Nyachoti, C. M. (2009). Nutritional quality of legume seeds as affected by some physical treatments, Part 1: Protein quality evaluation. LWT ‐ Food Science and Technology, 42, 1107–1112.

[fsn3473-bib-0019] Oomah, B. D. , Caspar, F. , Malcolmson, L. J. , & Bellido, A.‐S. (2011). Phenolics and antioxidant activity of lentil and pea hulls. Food Research International, 44, 436–441.

[fsn3473-bib-0021] Reyes‐Moreno, C. , Paredes‐López, O. , & Gonzalez, E . (1993). Hard to cook phenomenon in common beans—A review. Critical Reviews in Food Science and Nutrition, 33, 227–286. 8484867 10.1080/10408399309527621

[fsn3473-bib-0022] Sarwar, G. , & Peace, R. (1986). Comparisons between true digestibility of total nitrogen and limiting amino acids in vegetable proteins fed to rats. The Journal of Nutrition, 116, 1172–1184. 3746456 10.1093/jn/116.7.1172

[fsn3473-bib-0023] Savage, G. P. , & Thompson, D. R. (1993). Effect of processing on the trypsin inhibitor content and nutritive value of chickpeas (Cicer arietinum). Publication‐European Association for Animal Production, 70, 435–435.

[fsn3473-bib-0024] Tavano, O. L. , da Silva, S. I. , Demonte, A. , & Neves, V. A. (2008). Nutritional responses of rats to diets based on chickpea (Cicer arietinum L.) seed meal or its protein fractions. Journal of Agricultural and Food Chemistry, 56, 11006–11010. 18942847 10.1021/jf8010799

[fsn3473-bib-0025] Tzitzikas, E. N. , Vincken, J. P. , de Groot, J. , Gruppen, H. , & Visser, R. G. (2006). Genetic variation in pea seed globulin composition. Journal of Agricultural and Food Chemistry, 54, 425–433. 16417300 10.1021/jf0519008

[fsn3473-bib-0026] USDA . (2016). USDA Food Composition Database. Retrieved from https://ndb.nal.usda.gov/ndb/

[fsn3473-bib-0027] Vidal‐Valverde, C. , Frias, J. , & Valverde, S. (1992). Effect of processing on the soluble carbohydrate content of lentils. Journal of Food Protection, 55, 301–304. 10.4315/0362-028X-55.4.301 31071779

[fsn3473-bib-0028] Wang, N. , & Daun, J. K. (2005). Determination of cooking times of pulses using an automated Mattson cooker apparatus. Journal of the Science of Food and Agriculture, 85, 1631–1635.

[fsn3473-bib-0029] Wang, N. , Hatcher, D. W. , & Gawalko, E. J. (2008). Effect of variety and processing on nutrients and certain anti‐nutrients in field peas (Pisum sativum). Food Chemistry, 111, 132–138.

[fsn3473-bib-0030] Wang, N. , Hatcher, D. W. , Toews, R. , & Gawalko, E. J. (2009). Influence of cooking and dehulling on nutritional composition of several varieties of lentils (Lens culinaris). LWT ‐ Food Science and Technology, 42, 842–848.

[fsn3473-bib-0031] Wang, N. , Hatcher, D. W. , Tyler, R. T. , Toews, R. , & Gawalko, E. J. (2010). Effect of cooking on the composition of beans (Phaseolus vulgaris L.) and chickpeas (Cicer arietinum L.). Food Research International, 43, 589–594.

[fsn3473-bib-0032] Wu, W. , Williams, W. P. , Kunkel, M. E. , Acton, J. C. , Huang, Y. , Wardlaw, F. B. , & Grimes, L. W. (1996). Amino acid availability and availability‐corrected amino acid score of red kidney beans (Phaseolus vulgaris L.). Journal of Agricultural and Food Chemistry, 44, 1296–1301.

